# Silver spoon effects on plumage quality in a passerine bird

**DOI:** 10.1098/rsos.140459

**Published:** 2015-06-03

**Authors:** Piotr Minias, Radosław Włodarczyk, Adrian Surmacki, Tomasz Iciek

**Affiliations:** 1 Department of Teacher Training and Biodiversity Studies, University of Łódź, Banacha 1/3, 90-237 Łódź, Poland; 2 Deparment of Avian Biology and Ecology, Adam Mickiewicz University, Umultowska 89, 61-614 Poznań, Poland; 3 Rojna 37/21, 91-134 Łódź, Poland

**Keywords:** *Carduelis chloris*, greenfinch, feather quality, moult, plumage quality, silver spoon effect

## Abstract

A silver spoon effect means that individuals who develop under favourable circumstances enjoy a fitness or performance advantage later in life. While there is large empirical support for silver spoon effects acting on different life-history traits in birds, such as survival and reproduction, the evidence for the carry-over effects of rearing conditions on the quality of future plumage generations is lacking. Here, we examined whether abilities of individuals to undergo extensive post-juvenile moult may depend on the quality of juvenile plumage developed during the nestling phase in a small passerine showing large individual variation in the extent of post-juvenile moult, the greenfinch (*Carduelis chloris*). We found that high structural quality and carotenoid chroma of juvenile feathers were positively linked to the extent of post-juvenile moult in this species, thus allowing young birds to attain more adult-like plumage. Silver spoon effects mediated by the juvenile plumage quality were also found to have other fitness-related consequences, as individuals with high-quality juvenile feathers were in better condition during their first winter. As far as we are aware, the results provide the first correlative evidence for a silver spoon effect acting on general plumage quality in birds.

## 1. Introduction

Moulting is an important, but energetically demanding stage of the avian annual cycle. The process of moult is necessary to replace damaged or lost feathers in order to maintain basic functions of the plumage, but it also allows birds to adjust the plumage to the changing selective pressures over their lifetime [1]. The first moult after fledging (post-juvenile moult) usually allows transition from a distinct juvenile plumage developed in the nestling phase into the adult-like first-winter plumage. While the extent of post-juvenile moult is strictly specified and invariable in many avian species, a number of passerines show great individual variation in this trait [1]; however, this phenomenon has been largely neglected in the studies on the ecological causes and consequences of seasonal moulting.

As an extensive post-juvenile moult reduces juvenile plumage characters, it is likely to increase dominance rank and enhance competitive abilities [2]. Consequently, individuals with more adult-like plumage may have better access to food resources [3], thus increasing their probability of survival over the first winter period. It remains unanswered, however, whether ability to attain more adult-like plumage is primarily driven by the factors acting directly at the time of moulting or whether it may also be determined earlier in life. Here, we examined whether abilities of individuals to undergo extensive post-juvenile moult may be related to the quality of juvenile plumage developed during the nestling phase. For this purpose, we measured structural quality and carotenoid chroma of juvenile feathers and the quality of first-winter plumage (expressed as the number of adult-like feathers) in a small passerine showing large individual variation in the extent of post-juvenile moult, the greenfinch (*Carduelis chlori*s). Although structural quality of feathers is known to have a certain genetic component [4], there is also much evidence that it is strongly dependent on the availability of resources during feather production [5]. Carotenoid coloration of feathers is likely to be under even less genetic control (heritability *h*^2^<0.18, reviewed in [6]), as carotenoids are derived solely from diet, and environmental factors (most possibly food quality) have been reported to underlie the majority of variation in carotenoid-based plumage traits [7,8]. Thus, both structural quality and carotenoid chroma of juvenile feathers are expected to be largely determined by the conditions experienced at the nestling stage.

We have put forward two alternative hypotheses to explain potential relationships between the quality of juvenile and post-juvenile plumage: (i) individuals with low-quality juvenile plumage should allocate large energetic resources to increase the extent of post-juvenile moult, as it may be crucial in terms of survival to replace as many low-quality juvenile feathers as possible before winter; (ii) individuals with low-quality juvenile plumage are constrained energetically and moult less extensively than conspecifics with high-quality plumage. The latter hypothesis is consistent with the silver spoon effect, where individuals who develop under favourable circumstances enjoy a fitness or performance advantage later in life [9]. To assess whether the silver spoon effect acted on other fitness-related traits, we also tested for the relationships between the quality of juvenile plumage and two indices of first winter body condition, i.e. fat deposits and size-adjusted body mass. Although a number of life-history traits in vertebrates have been reported to be affected by rearing conditions [10,11], we are not aware of any empirical evidence for the silver spoon effect acting on general plumage quality in birds, except for specific sexually selected ornaments [12,13].

## 2. Material and methods

The fieldwork took place in the suburban area of Łódź (51°49′ N, 19°22′ E), central Poland, between the beginning of January and the end of April 2009. Greenfinches were captured at the baited feeders and during the entire period of study 605 young (2-calendar-year-old) individuals were caught. All birds were sexed by plumage and ringed. Wing length was measured with a stopped ruler to the nearest 1 mm and body mass with an electronic balance to the nearest 0.1 g. Winter body condition was calculated as the ratio of body mass to wing length, which is a common practice in avian studies [14,15]. Visible fat deposits in the furculum region and over the abdomen were assessed by one of the authors (T.I.) according to a 0–5 score scale developed for passerines [16] and square-root-transformed to improve normality. At the time of capture, none of the greenfinches was actively moulting, which indicated that all birds had already completed their post-juvenile moult. The presence of moulted feathers was examined in the following tracts: flight feathers (primaries, secondaries, tertials), wing coverts (primary and greater coverts) and tail feathers (rectrices). Juvenile and adult-like feathers were distinguished visually by age-specific coloration patterns. The extent of post-juvenile moult was expressed as the total number of moulted feathers in these tracts, ranging from 0 to 45 (53.4% of all evaluated feathers) per individual in the studied population (for details, see [17]).

### 2.1 Feather quality measurements

A pair of outermost rectrices was plucked from each captured bird. The total length of each feather (the distance from the calamus base to the distal feather tip) was measured twice with callipers (±0.01 mm) and averaged. Repeatability of the measurement calculated as an intra-class correlation coefficient [18] was 0.998. All feathers were also weighed to the nearest 0.01 mg on a digital balance. Structural quality of feathers was estimated from residuals of feather mass against length, which provides a size-independent measure of the structural complexity of feathers with positive residuals (high-quality feathers) showing a wider rachis and a greater density of barbs than negative residuals (low-quality feathers). Consequently, positive residuals are likely to reflect such properties of feathers as higher bending stiffness and resistance to wear [19]. As reported for other passerines, structural quality of juvenile tail feathers well correlates with the quality of other contour feathers, and thus may be used as a reliable proxy for the quality of entire juvenile plumage [20]. Feather quality was calculated only for individuals that retained both juvenile outermost rectrices (*n*=508).

### 2.2 Reflectance measurements

We measured reflectance of juvenile rectrices in 60 randomly selected greenfinches (30 individuals per sex). The measurements were conducted using a USB4000 spectrometer connected to a pulsed xenon lamp PX2 (Ocean Optics, Dunedin, FL, USA) with a bi-furcated fibre-optic measuring probe FCR-7UV200-2-1.5×100 (Avantes, Apeldoorn, The Netherlands). To avoid ambient light and to standardize measuring distance (1.5 mm), a brass tip was mounted on the ferrule of the probe. The probe was held at a 90° angle to the feather surface and illuminated an area of *ca* 1 mm diameter. Before measuring the feathers, we standardized measurements using a white standard (WS-1-SL, Labsphere, North Sutton, NH, USA) while the dark standard was taken by turning off the light source and covering the probe. Spectral measurements were expressed as per cent reflectance of light per wavelength in relation to a white standard reflectance (100%). We took four readings from the dorsal side of the left and right outermost rectrices of each bird, including two readings per each vane of the feather. In the inner vane, the measurements were taken from the standard position, *ca* 5 mm from the edge of the dark, melanin patch [21]. In the outer vane, we measured the widest part of the vane, just above the distal edge of the quill. We processed all the spectral data using RCLR v0.9.28 software [22]. For each individual, we calculated carotenoid chroma ((*R*_700_−*R*_450_)/*R*_700_; based on S9 in [22]), which is a good predictor of carotenoid content in yellow feathers [23]. In the analyses, we used carotenoid chroma averaged separately for the inner and outer vane of the feathers.

### 2.3 Statistical analyses

The effects of structural quality and carotenoid chroma of juvenile feathers on the extent of post-juvenile moult and winter body condition were tested with the general linear models. Box–Cox transformation was used to normalize the distribution of moult extent prior to the analysis. In all the models, the effect of sex was included as a fixed factor and an interaction between sex and the tested covariate was entered to check whether investigated relationships are similar for both sexes. All values are presented as means±s.e. and effect sizes were reported as partial *η*^2^ [24]. All statistical analyses were performed with Statistica v. 10.0 (StatSoft, Tulsa, OK, USA).

## 3. Results

**Figure 1.**
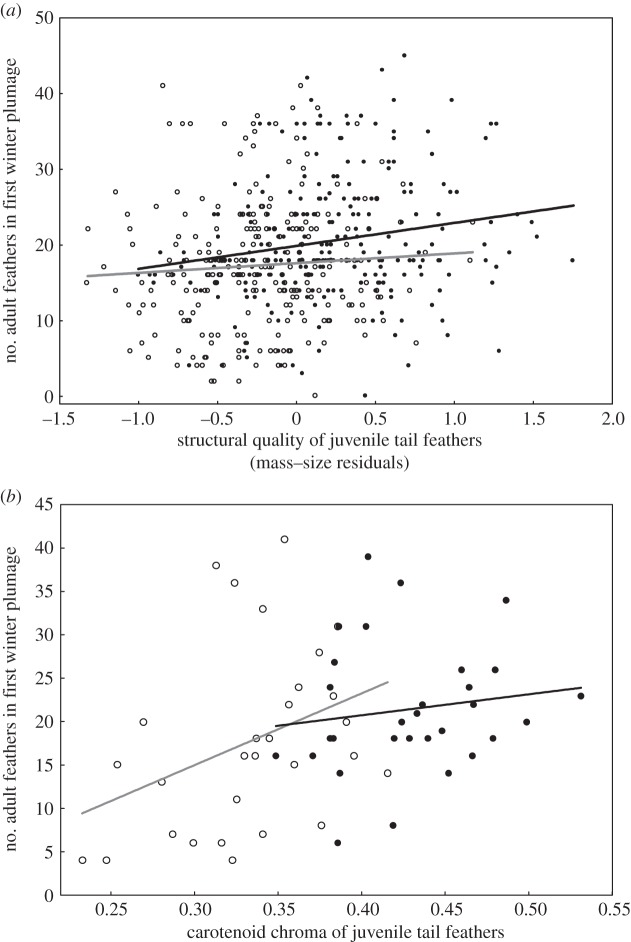
Extent of post-juvenile moult (the number of adult feathers in first winter plumage) in relation to the structural quality (*a*) and carotenoid chroma (*b*) of juvenile tail feathers in male (filled circles, black line) and female (open circles, grey line) young greenfinches. The effects of sex are non-significant. Lines indicate fitted regressions.

The extent of post-juvenile moult varied between sexes (*F*_1,506_=21.17, *p*<0.001), as males underwent more extensive moult in comparison to females (20.50±0.50 versus 17.21±0.52 moulted feathers). Male juvenile feathers were also of higher structural quality (measured as mass–length residuals) in comparison to female feathers (0.22±0.03 versus −0.24±0.03; *F*_1,506_=118.93; *p*<0.001). After accounting for the effect of sex, we found that there was a significant positive relationship between the quality of juvenile rectrices and the extent of post-juvenile moult (*F*_1,504_=7.69, *p*=0.006; *β*=0.26±0.09; figure 1*a*), although the quality of juvenile feathers explained only 1.5% of the variance in moult extent. The relationship was similar for both sexes as indicated by insignificant interaction between sex and feather quality (*F*_1,504_=0.89, *p*=0.34; figure 1*a*).

Post-juvenile moult extent was also associated with carotenoid chroma of the inner and outer vane of juvenile rectrices (CC_inner_: *F*_1,58_=8.90, *p*=0.004; CC_outer_: *F*_1,58_=4.51, *p*=0.038). Consistently with the results on feather mass–length residuals, individuals with higher carotenoid chroma of rectrices showed more extensive post-juvenile moult (CC_inner_: *β*=6.12±2.05; CC_outer_: *β*=5.21±2.46). However, after accounting for the sex variation in moult extent, its relationship with carotenoid chroma of the outer feather vane marginally lost significance (*F*_1,56_=2.86, *p*=0.096). By contrast, relationship of moult extent with carotenoid chroma of the inner feather vane remained significantly positive (*F*_1,56_=5.36, *p*=0.024, *β*=7.04±3.04; figure 1*b*) and it was independent of sex (CC_inner_–sex interaction: *F*_1,56_=1.64, *p*=0.21; figure 1*b*). Carotenoid chroma of the inner and outer vane of juvenile rectrices explained 13.3% and 7.2% of variance in the extent of post-juvenile moult, respectively. Nevertheless, after including the effect of sex in the model the per cent of explained variance was reduced to 8.9% and 3.3% for the inner and outer feather vane, respectively.

**Figure 2.**
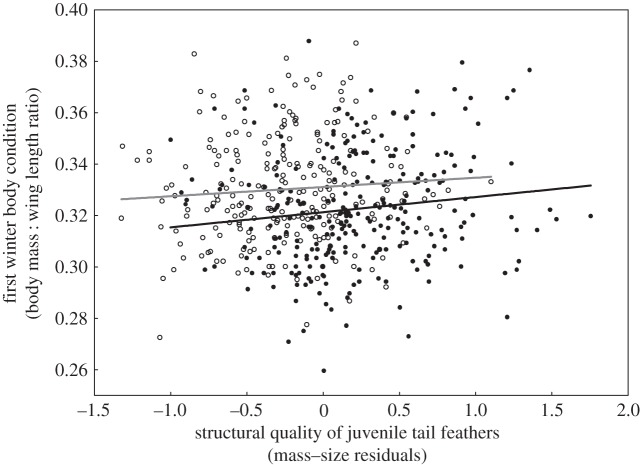
First winter body condition (body mass : wing length ratio) in relation to the structural quality of juvenile tail feathers in male (filled circles, black line) and female (open circles, grey line) young greenfinches. The effect of sex is non-significant. Lines indicate fitted regressions.

We also found support for silver spoon effects acting on body condition of young greenfinches during their first winter. There was a positive relationship of structural quality of juvenile tail feathers with first winter body mass : wing length ratio of greenfinches (*F*_1,503_=5.28, *p*=0.022, *β*=0.005±0.002, *η*^2^=0.01; figure 2), and this relationship was independent of sex (*F*_1,503_=0.31, *p*=0.57). Although carotenoid chroma of juvenile tail feathers was not related to body mass : wing length ratio during the first winter (CC_inner_: *F*_1,56_=0.31, *p*=0.58; CC_outer_: *F*_1,58_=0.02, *p*=0.89), we found a sex-dependent relationship between carotenoid chroma of the inner vane of juvenile rectrices and winter fat deposits (CC_inner_–sex interaction: *F*_1,56_=7.93, *p*=0.007). We found that carotenoid chroma of the inner vane was positively related with winter fat deposits in females (*F*_1,28_=6.12, *p*=0.020, *β*=3.49±1.41, *η*^2^=0.18), while no significant relationship was found for males (*F*_1,28_=2.07, *p*=0.16). Carotenoid chroma of the outer vanes of juvenile rectrices was not related to winter fat deposits in any of the sexes (*F*_1,56_=1.54, *p*=0.21).

## 4. Discussion

The results of this study indicated a silver spoon effect acting on plumage quality in a small passerine, the greenfinch, where structural quality and carotenoid chroma of juvenile feathers were positively associated with the extent of adult-like plumage during the first winter. We suggest that this pattern may be explained by a combination of two non-exclusive mechanisms: (i) direct impact of favourable rearing conditions affecting both the quality of juvenile feathers as well as post-fledging condition and the extent of post-juvenile moult; (ii) additive benefits of high-quality juvenile plumage gained in terms of better flight performance and thermoregulatory capabilities (higher structural quality of feathers) or social status (higher carotenoid chroma), which may increase post-fledging condition and facilitate more extensive post-juvenile moult. Unfortunately, our non-experimental data allow neither to distinguish between these mechanisms nor to determine whether the observed relationships are of causal or correlative nature.

So far, there is a large body of evidence for short-term silver spoon effects on juvenile survival [11,25–27] or natal dispersal [28–30], while empirical support for the long-term effects is scarcer [10,31]. This pattern suggests that although favourable rearing conditions may be beneficial throughout life, they are likely to be most pronounced during early life stages. Any potential long-term effects of rearing conditions may be limited by accumulating environmental stochasticity that individuals experience during life [5]. Non-exclusively, selection gradients of fitness components usually become less strong over lifetime, so parents may gain higher fitness pay-offs by enhancing short-term fitness prospects of their offspring [32].

Although we are not aware of any evidence for the silver spoon effects acting on the general quality of plumage, it has been reported that rearing conditions may affect specific sexual ornamentation in birds. Male collared flycatchers (*Ficedula albicollis*) recruited from nests in which brood size was experimentally reduced developed a larger secondary selected plumage trait, the white forehead patch [1]. In male barn swallows (*Hirundo rustica*), brood sex composition had long-term carry-over effects on the length of the outermost tail feathers, a condition-dependent trait under inter-sexual selection [13]. Similar effects of rearing conditions have been reported for non-plumage-based ornaments in birds [33,34]. As greenfinch shows no pre-breeding partial moult at wintering grounds, plumage attained in the post-juvenile moult may also be sexually selected in recruited individuals during their first breeding season, thus having certain reproductive consequences. In fact, within-individual changes in plumage traits have already been shown to correlate with reproductive performance in birds [35]. However, we found that the silver spoon effects mediated by the juvenile plumage quality had also fitness-related consequences on non-sexually selected traits, as individuals with high-quality juvenile feathers were in better condition during their first winter. This was consistent with the previous findings from the same greenfinch population indicating that first winter body condition was positively associated with the extent of adult-like plumage [17]. Thus, it seems probable that the plumage-mediated carry-over effects of rearing conditions may not be limited to sexually selected fitness components in this, and possibly in other avian species.

## Supplementary Material

Raw data

## Data accessibility

The datasets supporting this article have been uploaded as the electronic supplementary material.
